# [Retracted] miR-92a-3p promotes the proliferation, migration and invasion of esophageal squamous cell cancer by regulating PTEN

**DOI:** 10.3892/ijmm.2024.5453

**Published:** 2024-11-04

**Authors:** Xin Li, Shuilong Guo, Li Min, Qingdong Guo, Shutian Zhang

Int J Mol Med 44: 973-981, 2019; DOI: 10.3892/ijmm.2019.4258

Following the publication of this paper, a concerned reader drew to the Editor's attention that it appeared as if the authors had calculated the apoptotic rates erroneously.

The authors were asked to provide an explanation to account for the concerns raised by the interested reader; however, they did not respond to this request submitted by the Editorial Office. Therefore, owing to the lack of responsiveness on the part of the authors, the Editor of *International Journal of Molecular Medicine* has decided that this paper should be retracted from the journal. The Editor would like to apologize to the readership for any inconvenience caused.

